# Abdominal pain is a main manifestation of delayed bleeding after splenic injury in patients receiving non-operative management

**DOI:** 10.1038/s41598-022-24399-9

**Published:** 2022-11-18

**Authors:** Yu-Cheng Su, Chia-Yu Ou, Tsung-Han Yang, Kuo-Shu Hung, Chun-Hsien Wu, Chih-Jung Wang, Yi-Ting Yen, Yan-Shen Shan

**Affiliations:** 1 School of Medicine, College of Medicine, National Cheng Kung University, Tainan, Taiwan; 2 Department of Surgery, National Cheng Kung University Hospital, National Cheng Kung University, Tainan, Taiwan; 3 Division of Trauma, Department of Surgery, College of Medicine, National Cheng Kung University Hospital, National Cheng Kung University, No. 138, Sheng Li Road, Tainan, Taiwan; 4 Division of General Surgery, Department of Surgery, National Cheng Kung University Hospital, Tainan, Taiwan; 5 Institute of Clinical Medicine, College of Medicine, National Cheng Kung University, Tainan, Taiwan

**Keywords:** Gastroenterology, Medical research

## Abstract

Delayed bleeding is a major issue in patients with high-grade splenic injuries who receive non-operative management (NOM). While only few studies addressed the clinical manifestations of delayed bleeding in these patients. We reviewed the patients with high-grade splenic injuries presented with delayed bleeding, defined as the need for salvage procedures following NOM. There were 138 patients received NOM in study period. Fourteen of 107 patients in the SAE group and 3 of 31 patients in the non-embolization group had delayed bleeding. Among the 17 delayed bleeding episodes, 6 and 11 patients were salvaged by splenectomy and SAE, respectively. Ten (58.9%, 10/17) patients experienced bleeding episodes in the intensive care unit (ICU), whereas seven (41.1%, 7/17) experienced those in the ward or at home. The clinical manifestations of delayed bleeding were a decline in haemoglobin levels (47.1%, 8/17), hypotension (35.3%, 6/17), tachycardia (47.1%, 8/17), new abdominal pain (29.4%, 5/17), and worsening abdominal pain (17.6%, 3/17). For the bleeding episodes detected in the ICU, a decline in haemoglobin (60%, 6/10) was the main manifestation. New abdominal pain (71.43%, 5/7) was the main presentation when the patients left the ICU. In conclusion, abdominal pain was the main early clinical presentation of delayed bleeding following discharge from the ICU or hospital.

## Introduction

Approximately a quarter of blunt abdominal traumas result in splenic injury, the most frequently encountered solid organ injury^1^. Over the past two decades, non-operative management (NOM) has been recommended as the primary treatment choice for haemodynamically stable patients to preserve splenic function^2–5^. In Taiwan, the NOM rate of splenic injury increased from 56 to 73% in tertiary centres^3,5,6^. In addition, the success rate of NOM can be up to 90%^3,4,7^.

Close monitoring of patients with high-grade splenic injury receiving NOM is necessary, as this group of patients is at a higher risk of treatment failure^3,4,8,9^. The failure rate of NOM ranges from 16.7 to 25.0%, depending on patient conditions^9,10^, such as injury severity score > 24, splenic injury grade > 2, and age > 40 years^2,11–13^. The timing of NOM failure ranges from hours to weeks after an injury^14^and there are no factors that can be used to predict this accurately. Continuous monitoring is necessary, as the risk of bleeding remains even after discharge^2^. Therefore, identifying the signs and symptoms of bleeding after NOM is important for early detection, early haemostasis, and the prevention of unfavourable outcomes^14,15^. However, few studies have discussed the clinical manifestations of delayed splenic bleeding after NOM^14–16^.

Here, we conducted a review of the outcomes in patients with high-grade splenic injuries over a 10 years period. This study aimed to identify the clinical manifestations of bleeding in patients with a high-grade splenic injury who received NOM.

## Material and methods

### Study participants

We conducted a retrospective study to review the medical records of patients with high-grade blunt splenic injury at the National Cheng Kung University Hospital (NCKUH) from 1 January 2010 to 31 December 2020. This study was approved by our institutional review board of National Cheng Kung University Hospital (IRB No. B-ER-111-120) and the requirement for informed consent was waived. All methods were performed in accordance with the relevant guidelines and regulations. We recruited patients with high-grade splenic injury from the trauma registry data bank. The severity of the injury was defined using the American Association for the Surgery of Trauma (AAST) Organ Injury Scale. All patients with splenic injury grade ≥ 3 were defined as having a high-grade splenic injury and were included in this study. The exclusion criteria were age < 16 years and splenectomy performed within 6 h of presentation to the emergency room. Data were obtained on patient demography, outcome, clinical management of the splenic injury, haemodynamic status, and laboratory test results.

### Definition of non-operative management and delayed bleeding

Patients with high-grade splenic injury who did not undergo splenectomy within the first 6 h after presenting to the emergency department were considered to have received NOM. Delayed bleeding was defined in patients who received NOM initially and required a salvage procedure, including splenectomy or splenic artery embolization (SAE). Patients who underwent angiography after NOM to evaluate any potential bleeding but did not receive SAE were classified as having no delayed bleeding.

### Non-operative management for splenic injury at NCKUH

Most patients with splenic injuries received NOM in our hospital unless they presented with an unstable haemodynamic status (systolic blood pressure < 90 mmHg). Angiography was indicated when the patients had contrast extravasation on computed tomography (CT), high-grade splenic injury, transient hypotension with a rapid fluid response, and a persistent decrease in haemoglobin level. Patients with high-grade splenic injury who received SAE or had grade IV–V splenic injury without embolization were observed in the intensive care unit (ICU) for 24–48 h with real-time monitoring and regular measuring of haemoglobin levels every 6–8 h. Patients who developed an unstable haemodynamic status had an indication for laparotomy. A persistent drop in haemoglobin levels, usually > 2 gm/dL, still requiring transfusion in the ICU was an indication for angiography to evaluate the potential need for SAE if the haemodynamic status was stable. Then, the patients were observed in the ward if they did not have severe associated injuries. Once the patients had signs and symptoms of delayed splenic injury and bleeding in the ward, they underwent a CT scan to evaluate the presence of active bleeding or new haematoma in the peritoneal cavity. Repeat angiography was the first choice of salvage procedure unless the patient had an unstable haemodynamic status.

### Statistical analysis

Continuous variables were analysed by Student’s *t* test, and categorical variables were analysed by chi-square tests. *P-*value < 0.05 was considered statistically significant. Statistical analysis was performed using SPSS Statistics for Windows, Version 17.0. (SPSS Inc., Chicago, IL, USA).

### Ethical approval and informed consent

This study was approved by our institutional review board and the requirement for informed consent was waived.

### Presentation

This paper had been presented at the 2021 Annual meeting of Taiwan Surgical Association.

## Results

**Figure 1:**
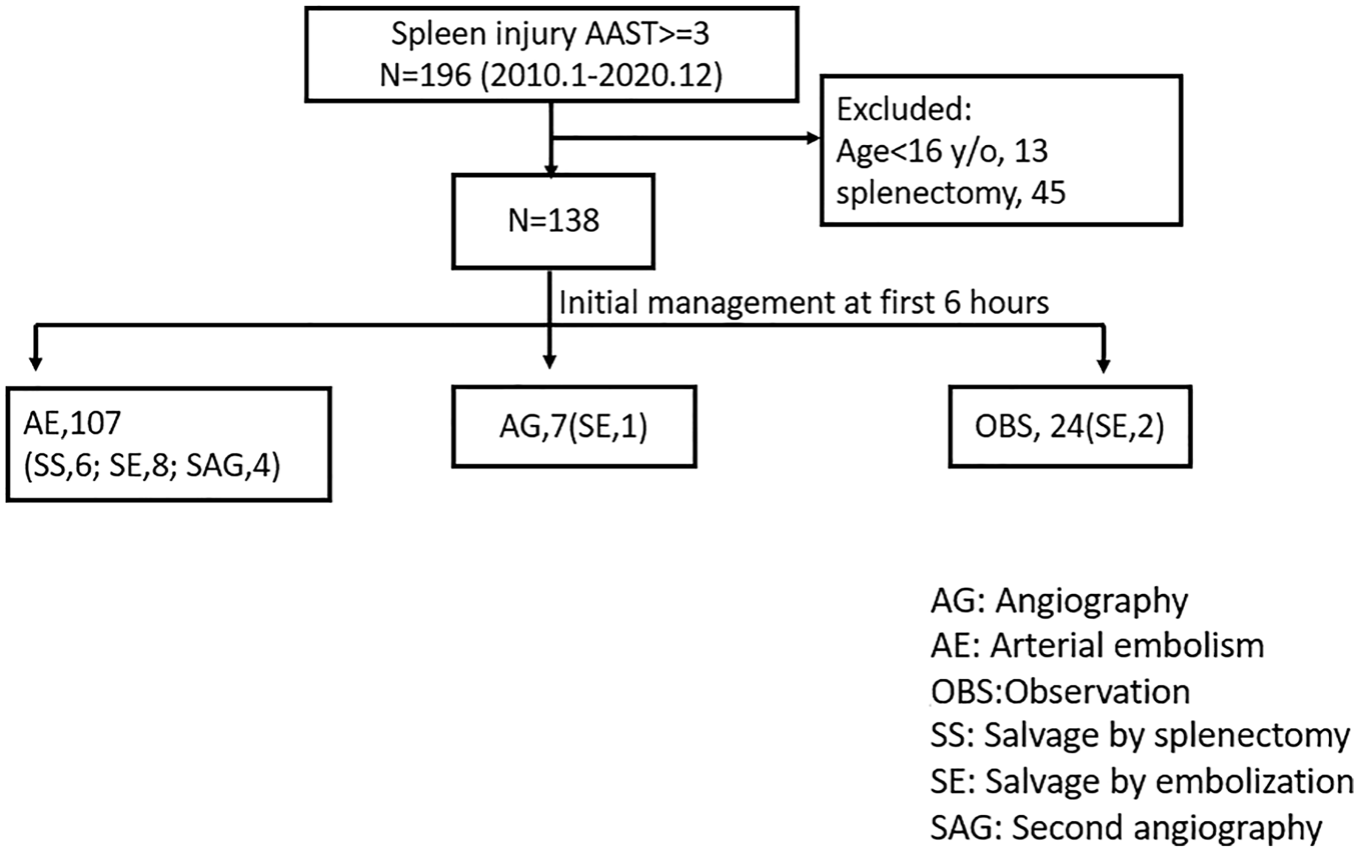
The initial management of patients following a blunt splenic injury. *AG* angiography, *AE* arterial embolism, *OBS* observation, *SS* salvage by splenectomy, *SE* Salvage by embolization, *SAG* second angiography.

A total of 196 patients presented with high-grade splenic injury. We excluded 13 patients aged < 16 and 45 patients who underwent splenectomy. Among the 138 patients who received NOM, 107 (77.5%) were initially managed with SAE and seven patients received angiography without embolization. Of the rest, 17 (12.3%) patients experienced bleeding episodes after NOM: 6 were salvaged by splenectomy and 11 by SAE. Four patients underwent angiography without embolization (Fig. 1).

There were no statistically significant differences in age, sex, and injury mechanism between the groups that received SAE and those that did not receive embolization (NE). The SAE group had more patients with shock index > 0.9 than the NE group, but this difference was not statistically significant (29.9% vs. 19.4%, *P* = 0.101). Compared with the NE group, the SAE group was associated with more severe haemoperitoneum (30.0% vs. 16.1%, *P* = 0.010), fewer grade III splenic injuries (43.9% vs. 74.2%, *P* = 0.012), and more arterial extravasation (47.7% vs. 25.8%, *P* = 0.030) (Table 1).

**Table 1:** Demographic characteristics of patients with blunt spleen injury that received NOM.

	Non-embolization (N = 31)	SAE (N = 107)	*P* value
Age (years), mean (SD)	41.74 (17.23)	35.74 (17.49)	0.094
Male (%)	20 (64.5)	85 (79)	0.098
**Mechanism (%)**			0.839
Traffic accident	25 (80.6)	93 (73)	
Fall	4 (12.9)	9 (8.4)	
Other	2 (6.4)	5 (4.6)	
Shock index > 0.9 (%)	6 (19.4)	32 (29.9)	0.101
**Heamoperitoneum (%)**			0.010
No	13 (41.9)	13 (12.1)	
Mild	9 (29.0)	37 (34.6)	
Moderate	3 (9.7)	26 (24.2)	
Severe	5 (16.1)	31 (30.0)	
**Spleen injury (AAST) (%)**			0.012
Grade 3	23 (74.2)	47 (43.9)	
Grade 4	5 (16.1)	42 (39.3)	
Grade 5	3 (9.7)	18 (16.8)	
Arterial extravasation (%)	8 (25.8)	51 (47.7)	0.030
Mortality (%)	0 (0)	1 (0.9)	0.589

*AAST* American Association for the Surgery of Trauma, *NOM* non-operative management, *SAE* splenic artery embolization *SD* standard deviation.

**Figure 2:**
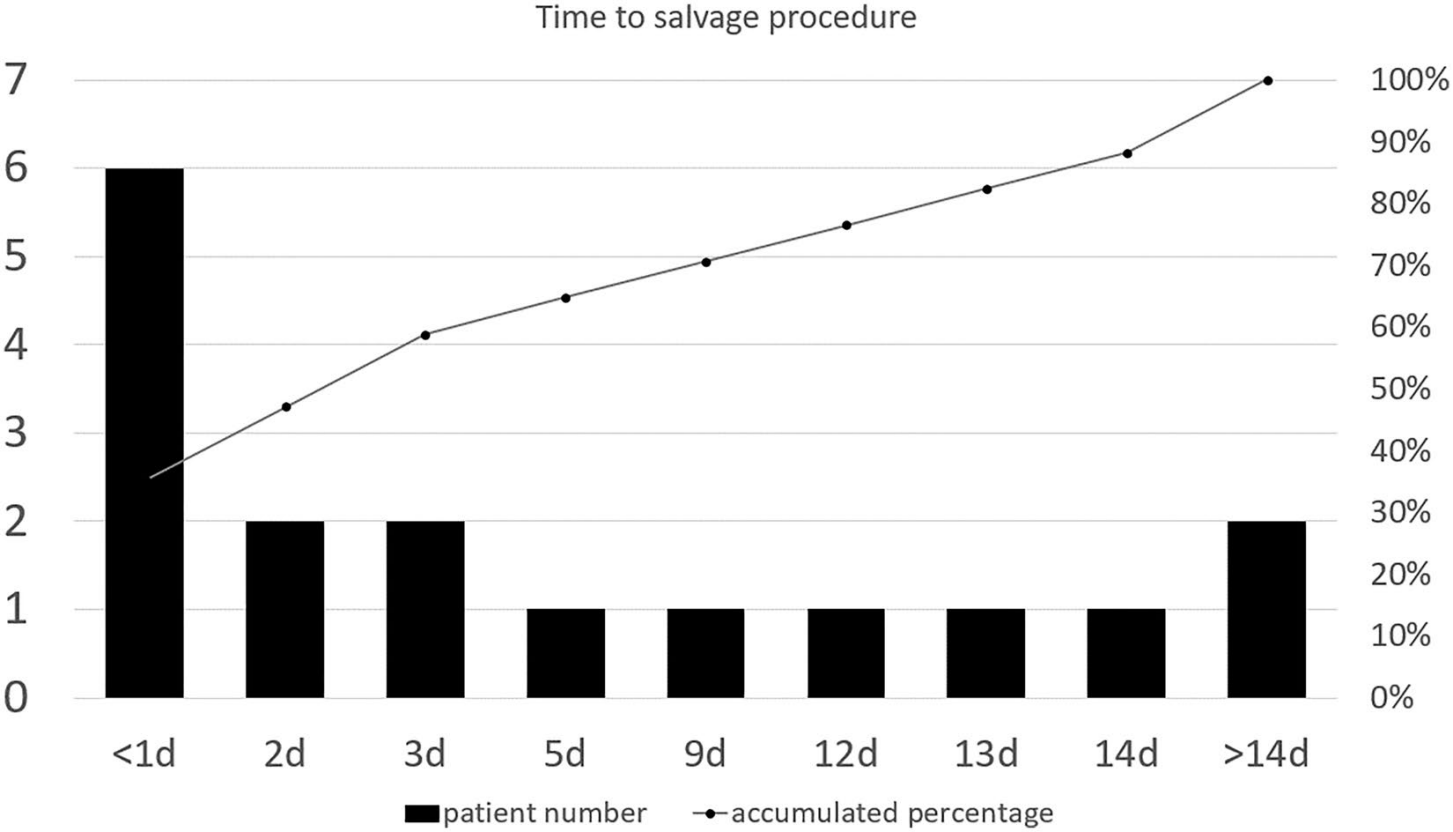
Time from admission to salvage procedure expressed as number of patients within each time period. Over 60% of the salvage procedures were performed within 5 days after injury.

For patients who had bleeding episodes after initial NOM, the time to the salvage procedure ranged from 8 h to 28 days (Fig. 2). Eleven (64.7%) salvage procedures were performed within 5 days after injury, while six patients (35.3%) received salvage procedures 9 to 28 days after injury. Six (35.3%) patients presented with blood pressure < 90 mmHg, eight (47.1%) experienced tachycardia (heart rate > 120 bpm, 4 h before salvage), and eight (47.1%) presented with decreased haemoglobin. Moreover, eight (47.1%) patients had the symptoms of abdominal pain: three (17.6%) presented with worsening and five (29.4%) with new abdominal pain. All patients who had bleeding episodes after NOM were successfully salvaged by either surgery or embolization in our hospital (Table 2).

**Table 2:** Characteristics of patients that underwent salvage procedures.

Age (years)	Group	Gra	TS	SP	Shock*	Tachy^†^	WAP	NAP	DHB
20	SAE	3	8 h	OP	N	Y	Y	N	Y
33	SAE	4	11 h	SAE	N	N	N	N	Y
37	SAE	3	15 h	OP	N	Y	Y	N	N
51	OBS	4	16 h	SAE	N	N	N	N	Y
59	SAE	4	1 day	OP	Y	Y	N	N	N
72	SAE	4	1 day	SAE	N	N	N	N	N
35	SAE	5	1 day 3 h	OP	Y	Y	N	N	Y
33	SAE	3	2 days	SAE	Y	N	N	N	N
19	SAE	3	2 days 12 h	SAE	N	N	N	N	Y
21	SAE	4	2 days 14 h	SAE	N	Y	N	N	Y
33	SAE	4	4 days 13 h	OP	Y	Y	N	N	Y
56	SAE	4	8 days 14 h	SAE	Y	N	Y	N	N
36	SAE	4	12 days	OP	N	N	N	Y	N
52	OBS	3	13 days	SAE	N	N	N	Y	N
27	SAE	4	14 days	SAE	N	Y	N	Y	N
44	OBS	4	16 days	SAE	N	N	N	Y	N
18	SAE	5	28 days	SAE	Y	Y	N	Y	Y
Total patients					35.3% (6/17)	47.1% (8/17)	17.6% (3/17)	29.4% (5/17)	47.1% (8/17)

*systolic blood pressure < 90 mmHg, ^†^heart rate > 120 beat/min.

*SAE* splenic artery embolization, *OBS* observation, *SBP* systolic blood pressure, *HR* heart rate *Hb* haemoglobin, *AAST* American Association for the Surgery of Trauma, *OP* operative management, *WAP* worsened abdominal pain, *NAP* new abdominal pain, *DHB* decreasing Hb, *C* Coil, *G* Gelfoam, *Tachy* tachycardia, *Gra* grade, *TS* time to salvage, *SP* salvage procedure, *EA* embolic agent, *N* No, *Y* Yes.

**Figure 3:**
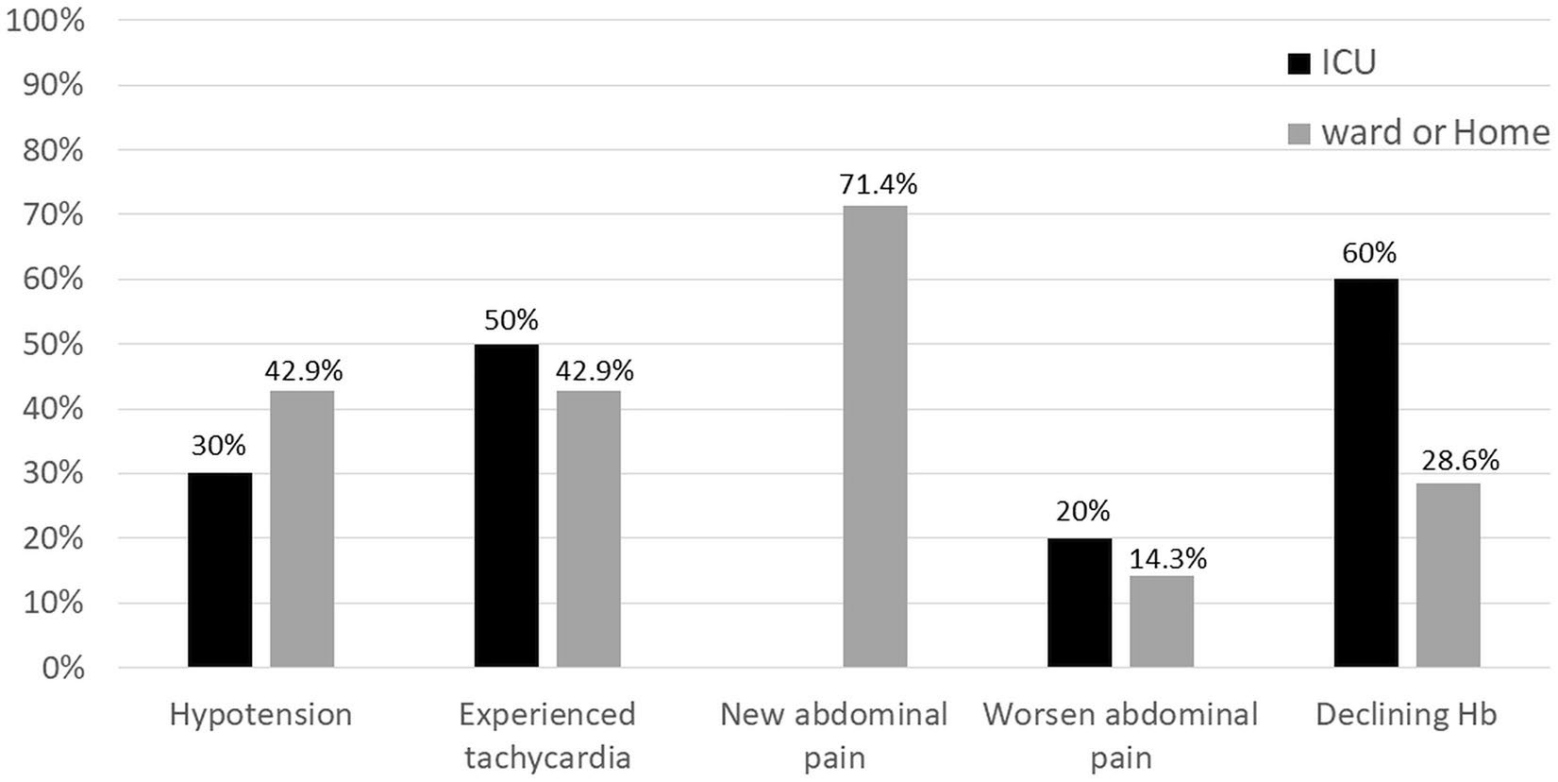
Clinical manifestations of delayed bleeding in patients in the ICU, in ward, and at home. Ten (58.9%) patients experienced delayed bleeding episodes in the ICU, while seven (41.2%) patients experienced delayed bleeding episodes in ward or at home. *ICU* intensive care unit.

Ten (58.9%) patients experienced bleeding episodes in the ICU, while seven (41.1%) patients experienced bleeding episodes when they were in the ward or at home. A decreased haemoglobin level (> 2 gm/dL) was the main manifestation of bleeding episodes detected in the ICU. New abdominal pain was the main presentation (71.4%) when a bleeding episode occurred in the ward or at home. When a bleeding episode occurred after day 9, all patients experienced abdominal pain (Table 2, Fig. 3).

## Discussion

This retrospective study analysed our experience with the management of delayed bleeding complications of high-grade blunt splenic injury after NOM. Seventeen of 138 (12.3%) high-grade splenic injuries required salvage procedures following NOM. Eleven and six patients were successfully salvaged by repeat SAE and splenectomy, respectively. Abdominal pain was the main manifestation of delayed bleeding for patients following discharge from the ICU. This is an important early warning sign of delayed bleeding from a splenic injury, especially as the patient leaves the hospital. To the best of our knowledge, few studies have reported on bleeding complications of splenic injury after NOM; these studies include 17 cases with delayed bleeding complications, only two of which included > 10 cases^14,17^. The current study demonstrated that abdominal pain is an important warning sign of delayed bleeding of splenic injury, especially in patients discharged from the ICU; this is an important manifestation that has rarely been emphasised in the literature.

Arterial embolization has been widely used for solid organ injury and can significantly increase the success rate of NOM for spleen injury^10^. According to recent studies, SAE resulted in > 90% success rate of NOM^3,4^. However, SAE could not totally prevent delayed bleeding episodes in patients receiving NOM. Approximately 17–25% of patients with splenic injuries had bleeding episodes after SAE^9^. Failure of NOM resulted in higher rates of mortality or prolonged hospitalisation^14^. However, there were no differences in the success rate of NOM or incidence of adverse events between patients who received prophylactic SAE and those who received indicated SAE^3^. In the current study, 17 of 138 (12.3%) patients needed salvage procedures after the initial NOM. Close monitoring of the signs and symptoms of bleeding is crucial for patients receiving NOM to detect bleeding episodes early and avoid unfavourable outcomes.

Delayed diagnosis of bleeding episodes can be dangerous or even deadly. Peitzman et al. reported 10 mortalities among 78 splenic injury patients with NOM failure^4,14,15,18^, and a 5–15% mortality rate have been reported by other studies^18–20^. Romeo et al. reported two cases of delayed splenic bleeding presenting with haemorrhagic shock, who were then admitted for several months^21^. Kodikara also reported a death due to delayed splenic rupture-related haemorrhagic shock^22^. Delayed bleeding episodes may result in acute haemodynamic instability, which significantly increases morbidity and mortality and is a risk factor for the development of multiple organ dysfunction^23^.

Most delayed bleeding episodes after the splenic injury occurred in the early period of NOM. Studies showed that 80–95% of splenic injuries had delayed bleeding episodes within 72 h of injury, and 18% of patients had failed NOM longer than 5 days after admission^4,14,24^. Thus, patients with high-grade splenic injury require close observation by real-time monitoring in the ICU. In our protocol, the patients were observed in the ICU for 24–48 h with regular monitoring of haemoglobin levels every 6–8 h after SAE. The bleeding episodes in our study occurred from 8 to 28 days after the start of NOM. Among patients who had bleeding episodes, 11 (64.7%) experienced them within 5 days, and 15 patients (88.2%) within 2 weeks of the injury. Several studies have suggested that longer observation periods should be adopted, but the need for observation eventually ended in up to 80% of patients within 14 days and in 95% within 21 days^24–27^. Ten (58.9%) patients experienced bleeding episodes in the ICU, while seven (41.2%) patients experienced bleeding episodes in the ward or at home. Most of the patients were discharged within one week. Prolonged observation in hospitals may not be practical and could waste medical resources. Accordingly, educating patients and their families about early signs of bleeding is relevant and crucial.

Abdominal pain may be a reliable manifestation of delayed bleeding in patients receiving NOM. In 1977, Olsen et al. demonstrated ten cases of delayed splenic rupture and showed that most of them experienced abdominal pain in different durations, patterns, and severity^17^. Farhat reported one case of delayed splenic rupture and mentioned that abdominal pain was common^28^. Peitzman et al. reported 78 cases of splenic injury with NOM failure, and the presentations of delayed bleeding in their study included haemodynamic decompensation (15%), decreased haemoglobin (36.5%), new abdominal pain (5%), worsening of abdominal pain (36.5%), and persistent tachycardia (16.2%)^14^. These were comparable with our results. According to the literature and our experience, the pattern of pain involved sudden onset of severe pain over the upper-left quadrant area^29^. Additionally, our study demonstrated that the main manifestations of delayed bleeding changed at different time periods (in the ICU, the ward or at home). Decreased haemoglobin levels were the main manifestation in patients requiring a salvage procedure in the ICU. However, new abdominal pain was the main presentation (71.4%) when a bleeding episode happened in the ward or at home. Only approximately one-third of patients with delayed bleeding had hypotension. For this reason, focusing on the signs of haemodynamic status may overlook patients with delayed bleeding. Here, three patients with delayed bleeding were unnoticed in the emergency department when they returned after discharge as they presented with symptoms of sudden abdominal pain rather than hypotension or a decline in haemoglobin levels. Recognising abdominal pain as one of the main presentations of delayed splenic bleeding is crucial for early diagnosis to avoid unfavourable outcomes. Additionally, Kofinas et al. mentioned the pattern of pain was left lower thorax and upper abdominal pain or tenderness in 2021^29^. It a similar clinical case of delayed splenic rupture, in which a young female received non-operative treatment initially similar clinical situation. Similarly, sudden onset of severe abdominal pain at left upper quadrant was the common pattern in our experience.

Currently, no definitive guidelines have been established for identifying delayed bleeding following a splenic injury that has been treated with NOM. The World Society of Emergency Surgery classification of splenic trauma and the management guidelines are evidence-based^2^ and recommended angiography, angioembolization, and splenectomy as salvage strategies for bleeding after NOM based on different indications. Operative management should be applied in cases of haemodynamic instability or if associated intra-abdominal injuries requiring surgical treatment are present^30^. The use of SAE for delayed splenic bleeding is supported by some evidence, for example, Liu et al. reported that five of six patients with delayed splenic bleeding were successfully salvaged by SAE^31^. Here, 17 of 138 (12.3%) patients received NOM for high-grade splenic injury and later needed salvage procedures, and 11 of the 17 patients with delayed bleeding were successfully salvaged by repeat embolization. Repeating SAE for selected patients with delayed splenic bleeding is a safe and feasible strategy.

The current study has several limitations. First, this was a retrospective study, and the patients’ symptoms and haemodynamic data were collected by reviewing their electronic medical records. Symptoms of abdominal pain could have been overlooked and incompletely recorded. However, haemodynamic data such as hypotension were less likely to be missed. Nevertheless, this did not affect our conclusion that abdominal pain was the main manifestation of delayed bleeding following discharge from the ICU. Second, some patients with delayed bleeding may have received treatment at other hospitals. We believe this probability to be low because most patients with a high-grade splenic injury received follow-up at our outpatient department, and it may not have affected the results of the current study. Third, the sample size was small as most previous studies were case reports. Only one study had more cases of delayed bleeding than the current study. Fourth, we frequently checked haemoglobin levels in the ICU and identified patients with signs of bleeding while the haemodynamic status was stable. Angiography usually shows oozing of the contrast in the spleen parenchyma. This type of bleeding may stop spontaneously, and our protocol may increase the incidence of delayed bleeding. However, the bleeding episodes only accounted for 12.3% of all the high-grade splenic injuries receiving NOM, which was not higher than that observed in previous studies^4,6,9,14,31^.

Delayed splenic bleeding is unpredictable and may occur within 4 weeks of the injury. The common clinical manifestations of delayed splenic bleeding include tachycardia, hypotension, a decline in haemoglobin levels, and abdominal pain. Abdominal pain is the main early clinical presentation of delayed bleeding after a patient leaves the ICU and hospital, and this is the first study to highlight its importance as an early clinical manifestation of delayed bleeding. Emphasising the importance of abdominal pain as an alarming presentation of delayed bleeding can help patients determine when to return to the hospital for timely management.

## Data Availability

The data that support the findings of this study are available from National Cheng Kung University Hospital, but restrictions apply to the availability of these data, which were used under license for the current study, and so are not publicly available. Data are however available from the authors upon reasonable request and with permission of correspondence.
